# Identifying Recall Under Sedation by a Novel EEG Based Index of Attention—A Pilot Study

**DOI:** 10.3389/fmed.2022.880384

**Published:** 2022-04-14

**Authors:** Dana Baron Shahaf, Avi Weissman, Leonid Priven, Goded Shahaf

**Affiliations:** ^1^Department of Anesthesia, Rambam Health Care Campus, Haifa, Israel; ^2^The Applied Neurophysiology Lab, Rambam Health Care Campus, Haifa, Israel

**Keywords:** EEG, attention, recall, anesthesia, sedation

## Abstract

**Overview:**

Recall is an accepted consequence of sedation. But due to the very low prevalence of the more devastating awareness under anesthesia (AUA), it might be of value to assess first the efficacy of new markers for AUA by their efficacy in discovering the more prevalent recall under sedation (RUS). In this pilot study we assessed whether a novel index for attentional effort, the cognitive effort index (CEI), derived in real-time from one forehead EEG channel, could differentiate between patients with or without RUS.

**Methods:**

EEG was sampled from 2 groups: (1) Patients who underwent deep sedation (*n* = 25) (using drugs according to the anesthesiologist preference, but generally combining either Midazolam or Propofol together with either Fentanyl or Remifentanil). (2) Patients who underwent general anesthesia (GA, *n* = 13, a negative control for recall). In recovery, recall was assessed using the BRICE questionnaire.

**Results:**

Of the 25 sedated patients, 11 experienced recall. The CEI marker was high during significantly longer periods in patients with recall, compared to sedated patients, or patients under GA, without recall. Moreover, the increase in CEI was evident mainly toward the end of the procedure.

**Conclusion:**

RUS seems to associate with higher level of attention, which is identified in real-time by the easy-to-extract CEI marker.

## Highlights

- This work demonstrates that monitoring an electrophysiological marker of attention can identify awareness under sedation.
- The marker is based on one forehead channel and is not hindered by muscle activity, which is prevalent under sedation.
- Our findings suggest that recall is more prevalent for attention evoking events which occur toward the end of the procedure.

## Introduction

Recall of awareness under anesthesia (AUA) is a serious adverse event with acute and long-lasting impact on patients (1–7). Various EEG-based markers have been developed in order to identify this condition during anesthesia (8).

It might be possible to differ in the literature among three major groups of electrophysiological markers, which are suggested for monitoring of consciousness under anesthesia. These include (i) ratios between the frontal activity in higher frequency bands and lower frequency bands, which are expected to increase between unconsciousness and consciousness (9); (ii) the degree of irregularity of the frontal activity, which is also expected to increase between unconsciousness and consciousness (10); and (iii) anteriorization of certain types of activity, and mainly of alpha activity, which is associated with loss of consciousness (11).

However, over recent years, significant evidence casts doubt regarding the ability of such markers to identify patients at risk for AUA with high precision, in part due to lack of sensitivity to various hypnotic agents (12) or the confounding influence of muscle activity, on the EEG signal (13–16). Thus, the recommendations include inspection of the EEG waves by the anesthesiologist, whenever processed EEG (pEEG) monitors are used during general anesthesia under total IV anesthesia (TIVA) (17). Therefore, there is certainly a need for further research and development of new and potentially more robust AUA markers. However, the very low prevalence of awareness under general anesthesia (GA) (5, 18–21) makes it difficult to validate the effectiveness of these markers.

Alternatively, sedation often involves greater prevalence of recall (5, 22, 23). The impact of recall under sedation (RUS) is usually not distressing, as patients are expecting some degree of awareness and are not paralyzed. Thus, they can signal when becoming aware and/or uncomfortable (5, 6, 24). Nevertheless, there are certainly procedures and conditions in which it is of clinical importance to monitor the patient's level of awareness under sedation (25–29) and multiple studies aimed at this goal, with limited success (30–32).

But for the purpose of developing markers for AUA, assessing RUS provided us with a study population with an expectedly higher incidence of recall, without impeding the impact of neuromuscular activity on the EEG signal, and enables an effective first evaluation of markers for awareness.

It is reasonable to expect that awareness under anesthesia or sedation, especially of stressing events, might evoke greater activation of attentional processes (32). It was shown, e.g., by using an auditory oddball protocol, that even though attention related electrophysiological activity reduces with increased dose of multiple anesthetics, it nevertheless remains evident. See, for example, the literature regarding: propofol (33), ketamine (34), nitrous oxide (35), midazolam, fentanyl, and thiopental (36).

Therefore, real-time markers for attention are expected to be effective for the identification of AUA and RUS. The literature contains well-established electrophysiological markers for attention. However, they are often based upon multi-channel EEG systems with tens of electrodes and are derived from long samples of data, often at the scale of many minutes (37). Therefore, such markers might be impractical, or too cumbersome, for real-time clinical use.

Recently we developed and validated real-time electrophysiological markers for attention, which are extractable from a single prefrontal EEG channel (38–44). Our markers of attention, the Brain Engagement Index (BEI) and the Cognitive Effort Index (CEI) (45), provide real-time values every 10 s. In accordance with the literature of attention-related markers (46), our markers tend to be low in conditions such as attention-deficit and depression, while they tend to be high in other conditions, such as anxiety and stress (39, 42). Furthermore, the markers were shown to tend toward a middle range with effective patient attention (38, 43). Thus, these markers seem to be valid and easy to use for real-time monitoring of attention.

In this pilot study, our objective was to test whether the CEI marker can be used in identifying recall under sedation. We previously showed a marker for RUS, but it was too cumbersome to use with multiple electrodes above hairline, with a poor signal to noise ratio (47). The use of CEI, which is easily extracted from below hairline electrodes, and has a good signal-to-noise ratio, was intended to overcome these limitations.

## Methods

### Participants

The study was approved by the Institutional Review Board (IRB), and written informed consent was obtained from all subjects. As an expansion of a previous study (47), we sampled additional 38 patients at Rambam healthcare campus, Haifa, Israel. Patients were recruited if they were: (1) undergoing sedation for various procedures (liver chemoembolization, biliary duct drainage); (2) were undergoing abdominal surgery under general anesthesia (GA). Exclusion criteria included patient age (<18 years), pregnancy and ASA IV-V.

### Study Flow

After patient recruitment, EEG was recorded during the procedure (which was done under sedation or GA), from its beginning until its end. For patients under GA, the EEG was placed after the patient's intubation. For patients under sedation, the EEG placement was done while the patient was connected to all other standard ASA monitoring. At the end of the surgery/procedure, when patients were fully awake at post anesthesia care unit (PACU), before discharge, they were assessed for having recall, using the standard Brice questionnaire (20, 48). The study was observational and the index was only analyzed offline.

### Sedation/Anesthesia Protocol

In the sedation group, patients were deeply sedated in accordance with an observer assessment alertness sedation (OAAS) scale of 0–1, using mostly combinations of either Midazolam and Fentanyl, Propofol and Fentanyl, or Midazolam and Remifentanil. This level of sedation was generally maintained stable throughout the procedure. Patients who underwent sedation were divided to two groups; with and without recall. Patients who underwent GA received balanced anesthesia, with no evidence of recall. The ranges of medication that were used for GA induction were: Propofol (1–2 mg/kg), Fentanyl (1–2 mcg/kg), and Rocuronium (0.6–1 mg/kg). After intubation, anesthesia was maintained with Sevoflurane (1 MAC).

### EEG System

EEG was sampled by Emotiv Epoc 128 Hz system (https://www.emotiv.com/epoc/). Data was sampled only from channel FP1 and referenced to T7, which are below hairline.

### Assessment of Recall Using the Brice Questionnaire

When patients were fully awake, before discharge from PACU, they were evaluated for recall using the Brice questionnaire (20, 48). The questionnaire included 3 main questions: (1) What is the last thing you remember before going to sleep? (2) What is the first thing you remember after waking up? (3) Do you remember anything between going to sleep and waking up? If patients answered “YES” to the third question, they were further questioned which type of recall they have (i.e., hearing events during the procedure, feeling anxiety or stress, feeling pain, feeling the procedure without pain) and were included in the recall group. If the patients answered “NO” to the third question, they were included in the no-recall group.

### CEI^1^ Analysis

Previously we computed the Brain Engagement Index (BEI) based on template matching at the delta band pass (1.5–4 Hz) (38–43, 49). The computation was based on measuring the number of occurrences of a pattern, which is composed from a sequence of large waves, lasting a few hundred milliseconds, followed by a sequence of small waves, also lasting a few hundred milliseconds. However, we recently found out it is not the precise pattern of waves that matters. Rather, it is the variability between epochs of greater delta power and epochs of less delta power (45).

**Figure 1:**
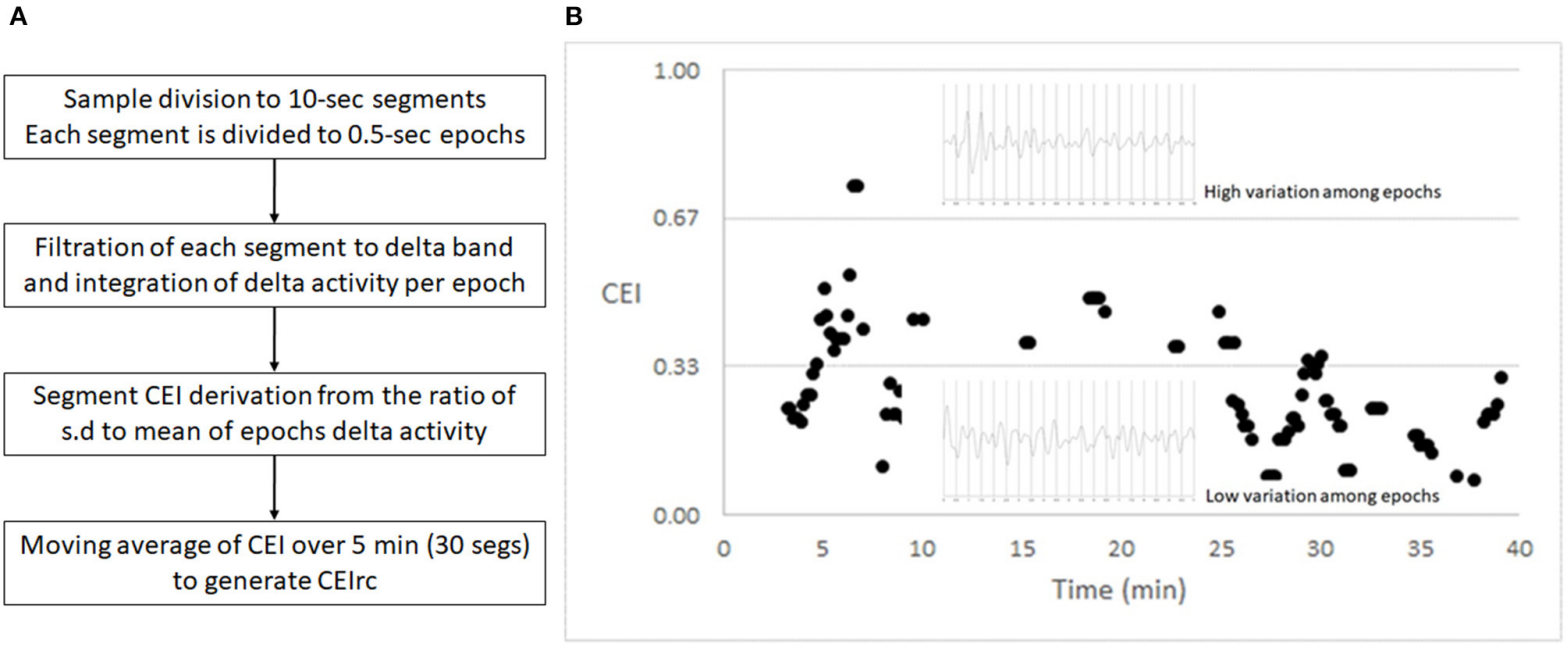
CEI – an index of variability. **(A)** The flow of CEIrc computation. **(B)** A 40 min sample of CEI values and two representative 10 s EEG segments, filtered to the delta band pass. Each of the segments is divided to 20 half-second epochs. The upper segment shows greater variability among the power of the half-second epochs and therefore generates a higher CEI value. The lower segment shows lesser variability among the power of the half-second epochs and therefore generates a lower CEI value.

Each 10-s segment was filtered to the delta band, and then the filtered segment is divided to 20 epochs of 500 ms each. For each epoch we computed the power of delta activity, and then we computed the mean and standard deviation of all epochs within a segment. Then the index was derived from the standard deviation: mean ratio and is in the [0,1] range. We learned that if the ratio is >1, it is likely to be due to a noisy sample, in which case no value was returned for this 10 s segment. The CEI was then averaged with moving windows of 5 min to generate the CEIrc (CEI for reduced consciousness)—See Figure 1 for a summary and an example of the CEIrc computation. We then compared among samples by the percent of time in which CEIrc was below a threshold (0.6).

As we described before (40), while this approach reduced overt noise, especially when prefrontal recording is considered, there is always concern regarding the impact of “milder” EMG/EOG noise sources, and especially, in the delta band pass in this region, there is concern regarding the effect of blinking (50). There seems to be a range of overlap in which it is uncertain whether activity originates from the brain or from muscle activity. However, interestingly, blinking is well-related to attention (51). Furthermore, it seems the pattern of “attentive” well-deferred blinking in the delta band pass may lead to greater variability of the signal, which would also be captured by the CEI marker (51). Therefore, we did not see a practical need to differentiate between EEG activity and blinking.

The CEI was developed to overcome the susceptibility of the BEI to noise (45). For example BEI tends not to return value (report as a type of noise) when the EEG activity is low (as is the case under anesthetics). Therefore, we used the more advanced CEI in this study. Nevertheless, it should be remembered that most of our validation studies were done with the BEI^2^.

### Statistical Analysis

Following statistical consultation, the comparisons of CEIrc among the study arms were evaluated with the Kruskal-Wallis one-way analysis of variance on ranks (with Bonferroni correction for multiple pairwise comparisons).

## Results

Twenty-five patients underwent sedation for various procedures in the angio room (biliary drainage insertion and liver chemoembolization) and 13 patients underwent GA for abdominal surgery. None of the patients in the GA group showed recall. The GA group age was 69 ± 10 years (mean ± s.d.) with a female: male ratio of 6:9. Eleven of the patients who underwent sedation showed recall (the recall group). The recall group age was 57 ± 13 years (mean ± s.d.), with a female: male ratio of 4:7. Fourteen of the patients who underwent sedation did not show recall. The no-recall age was 75 ± 36 years (mean ± s.d.), with a female: male ratio of 4:10.

### CEIrc in Patients With or Without Recall Under Anesthesia (Sedation or GA)

**Figure 2:**
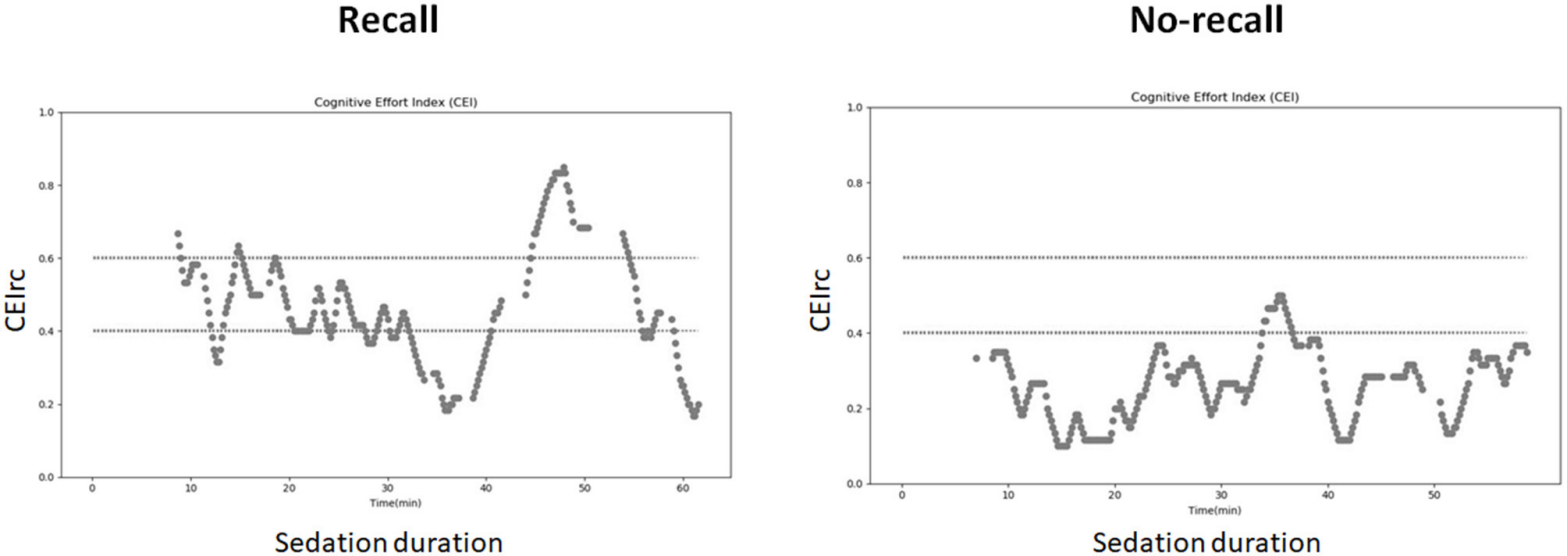
CEIrc dynamics under sedation. Left: a patient with recall; Right: patient without recall.

**Figure 3:**
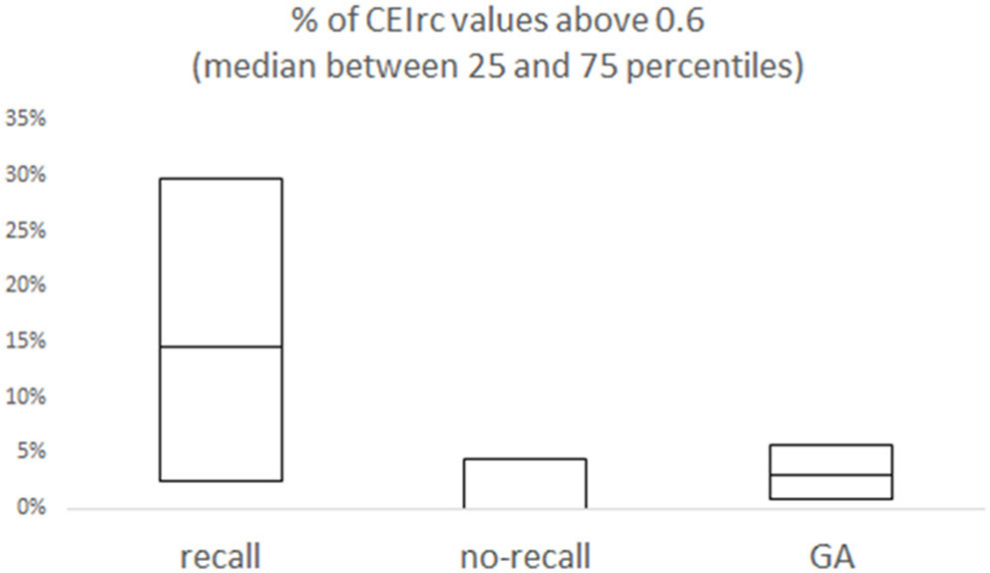
Overall % of CEIrc above the 0.6 threshold as a function of recall. For each group (sedation with recall; sedation with no-recall; GA with no-recall) the median and the inter quartile range (25–75%) are presented.

Representative examples of CEIrc dynamics under sedation in a patient with and a patient without recall are presented in Figure 2. As opposed to the index in our previous study (34), CEIrc values were robust to noise and were calculated throughout about 90% of the sample duration. The median time CEIrc was above the 0.6 threshold in patients having recall under sedation was 15% (inter quartile range, IQR, 2–30%). For patients under sedation without recall, the median time CEIrc was >0.6, was 0% (IQR 0–4%) and for patients under GA without recall it was 3% (IQR 1–6%), *p* < 0.05 (Figure 3). The difference was evaluated by Kruskal-Wallis one-way analysis of variance on ranks and was found significant, H (2)≈6.98, *p* ≈ 0.03. Pairwise comparison with Bonferroni correction for multiple comparisons revealed a significant difference only between the recall and no-recall groups (*p* ≈ 0.03). No significant differences were found between the GA group and the no-recall or recall groups (*p* ≈ 0.83 and *p* ≈ 0.35, respectively).

**Figure 4:**
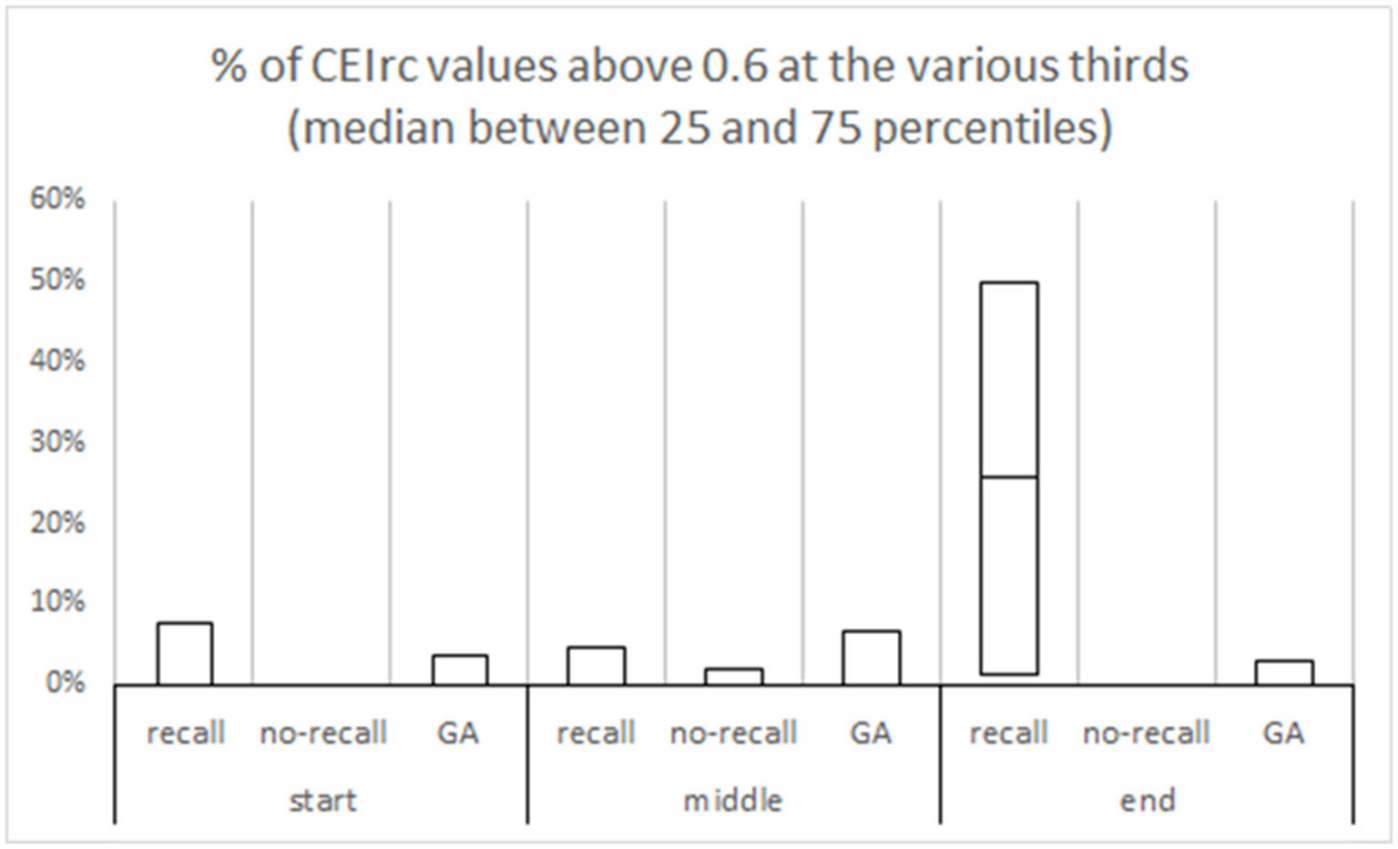
% of CEIrc above the 0.6 threshold in different parts of the procedure. Each procedure duration was divided to three equal parts—start, middle and end. In each part, the median and the inter quartile range are presented for each group (sedation with recall; sedation with no-recall; GA with no-recall).

We further divided the procedure to three parts (start, middle, and end) and evaluated the time the CEIrc was above the 0.6 threshold during each part. The median percentage of time CEIrc was above 0.6 in sedated patients with recall, during the end part of the procedure, was 26% (IQR 1–50%) compared to sedated patients without recall 0% (IQR 0–0%) and patients under GA without recall 0% (IQR 0–3%) (Figure 4). The differences in the three parts were also evaluated by Kruskal-Wallis one-way analysis of variance on ranks. There were no significant differences in the start and middle parts of the procedure, first part—H (2)≈1.78, *p* ≈ 0.41; second part—H (2)≈0.19, *p* ≈ 0.91. There was a significant difference in the third part—H (2)≈9.86, *p* ≈ 0.007. Pairwise comparison with Bonferroni correction for multiple comparisons revealed a significant difference between the recall and no-recall groups (*p* ≈ 0.02) and between the recall and GA groups (*p* ≈ 0.02). No significant difference was found between the no-recall and GA groups (*p* ≈ 0.39).

## Discussion

The underlying hypothesis, which was confirmed in a preliminary manner in this pilot study, was that awareness and recall under anesthetics would involve increased activation of attentional processes. We presented this hypothesis and reviewed its support in the literature, in much detail, in a previous theoretical publication (32). Electrophysiological markers for attention are well-established in the literature (37), and we established the ability to extract them in real-time from easy-to-use headsets (38–45).

While this is only a pilot study, it is nevertheless a first demonstration of the applicability of monitoring awareness and recall under anesthetics, using such an attention related marker. If these findings would be replicated, in more elaborative studies, they may offer a refreshing alternative to the currently prevailing markers of AUA and RUS, which are empirical in nature, and are considered imprecise (8). Specifically, the currently leading markers seem less appropriate for certain anesthetic drugs (52, 53), while attention, and attention related markers, seem to be reduced with all anesthetic drugs (32) and thus may overcome the limitations with the current markers.

An interesting finding emerging from this study is that timing of increase in attention matters, and recall is associated with enhanced attention toward the end of the procedure. Specifically, the no-recall groups differed significantly from the recall group only during the third part of the operation. This might mean that attention evoked by discomforting events during earlier stages of the operation, might be later wiped out by the amnestic effect of anesthetics (54). This finding could be applicable, as in cases of monitoring enhanced attention during surgery, it might be possible to consider using drugs with greater amnestic effect (54) and even to consider elongating the duration of anesthesia.

Monitoring RUS seems to have some potential clinical importance (25, 27, 31). However, a major interest would be to demonstrate the applicability of the CEIrc also for general anesthesia, which would require a huge sample size, or a daring study protocol (8). But as a first step in establishing a marker for AUA, it seems that validating the marker for sedation in a larger and prospective study would be of value. A major difference between sedation and general anesthesia would be the impact of muscle movement upon the signal in sedation, which is, at-least, greatly reduced under general anesthesia.

Beyond the need to evaluate the CEIrc directly for AUA, there are major limitations with the current study, which require further validation. The major limitation of the current study seems to be the small sample size. The other major limitation involves the use of a very limited set of anesthetic drugs—mainly midazolam and propofol. Therefore, even for sedation, these results need to be duplicated with a larger and more versatile sample, in a prospective study. Once the results are established in further studies, they could be expanded to other clinical populations, such as pediatric anesthesia, etc.

## Conclusion

We have provided data to support the hypothesis that a novel easy-to-use EEG index of attention, the CEIrc, might be applicable for monitoring RUS and potentially also AUA. An effective marker for attention might overcome the limitations of the currently prevailing markers for AUA, as attention is generally reduced with all anesthetic drugs. Further studies are required, to validate this index and to assess its value as a real-time clinical monitor.

## Significance

This work demonstrates for the first time the feasibility of using an easy-to-extract electrophysiological marker of attention for monitoring recall of awareness under sedation. Our findings suggest that recall might be more prevalent for attention evoking events which occur toward the end of the procedure.

## Data Availability Statement

The raw data supporting the conclusions of this article will be made available by the authors, without undue reservation.

## Ethics Statement

The studies involving human participants were reviewed and approved by Ethic committee of Rambam Health Care Campus. The patients/participants provided their written informed consent to participate in this study.

## Author Contributions

DBS, AW, and LP collected the data. GS developed the EEG marker. All authors reviewed the manuscript and approved it. All authors wrote the main manuscript text andfigures.

## Funding

This study was partially supported by Grant from the Israel Society of Anesthesia (ISA).

## Conflict of Interest

GS and DBS are spouses. GS founded several companies in the field of EEG analysis and DBS consulted one of these companies up to ~1 year ago. However, the present study is not related to these companies and their technology. It is based on developments in GS academic laboratory at Rambam Health Care Campus. The remaining authors declare that the research was conducted in the absence of any commercial or financial relationships that could be construed as a potential conflict of interest.

## Publisher's Note

All claims expressed in this article are solely those of the authors and do not necessarily represent those of their affiliated organizations, or those of the publisher, the editors and the reviewers. Any product that may be evaluated in this article, or claim that may be made by its manufacturer, is not guaranteed or endorsed by the publisher.

## Footnotes

^1^CEI is available to the interested researchers. See for example: https://store.neurosky.com/products/cei-monitor, or alternatively, contact the corresponding author of this paper.

^2^As our previous studies in this field validated the BEI ^40, 43, 44^, we compared the two indices, one to the other, during multiple samples of various clinical populations. BEI was found to be noisy in about 38% of the sample points, while CEI was found to be noisy in only about 6% of the sample points. While there are value differences between the two indices, there was 88% similarity in the dynamics of the two indices ^47^.

## Abbreviations

RUS: Recall Under Sedation
CEI: Cognitive Effort Index
GA: General Anesthesia
EEG: Electroencephalogram
AUA: Awareness Under Anesthesia
PACU: Post-Operative Care Unit
BEI: Brain Engagement Index
CEIrc: Cognitive Effort Index of reduced consciousness
IQR: Inter Quartile Range.
