# Supplementary material for: Identifying Recall Under Sedation by a Novel EEG Based Index of Attention—A Pilot Study
Source: Front Med (Lausanne). 2022 Apr 14;9:880384. doi: 10.3389/fmed.2022.880384 (PMC9047181; doi:10.3389/fmed.2022.880384)
Supplement: Supplementary file 1 [file Data_Sheet_1.PDF]

Recall

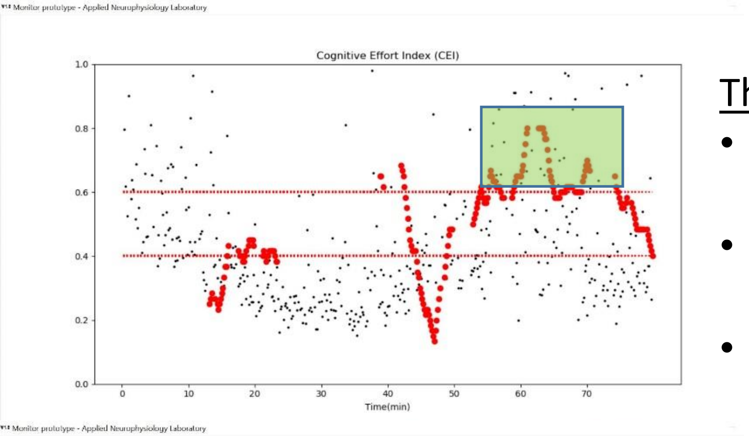

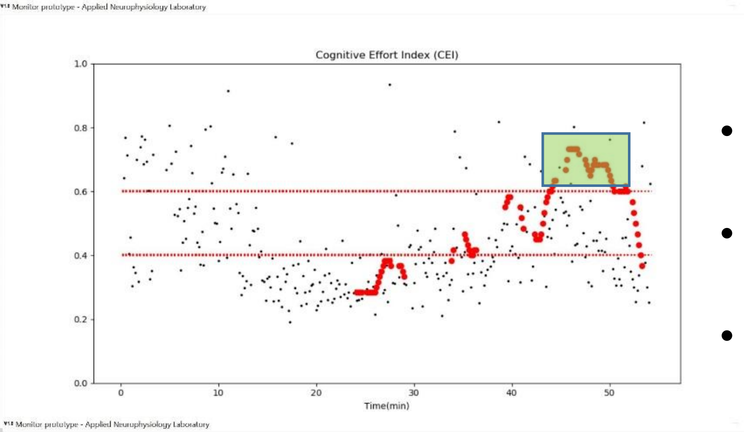

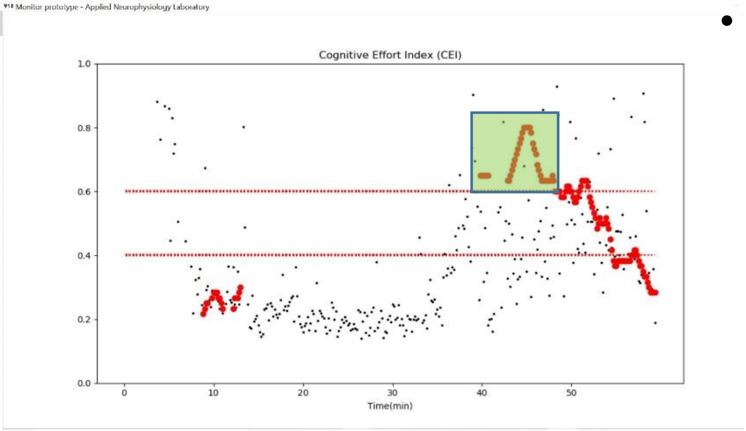

<u>TheCEIrc plots of the 25 patients who underwent sedation</u>

- The plots are divided to patients with and without recall (presnted in the header of each page)
- The CEIrc is presented in red (missing points are due to automatic rejection as noise)
- Segment delta power is presented in black, normalized to the [0,1] range by dividing each segment power by the maximal power, taken from the patient with the largest delta power segment
- Thus, all power values, across patients, are normalized to the same global value
- Periods in which CEIrc was above the 0.6 threshold in the last sample third are marked with a light green rectangle
- In each group, the samples are ordered by CEIrc, mainly during the last third of the operation
- Note that in the Recall group 7/11 patients reached values above the 0.6 threshold in the last third, for two others the sample in this period was too noisy, and the other two seemed to have reached their peak activity earlier

No recall

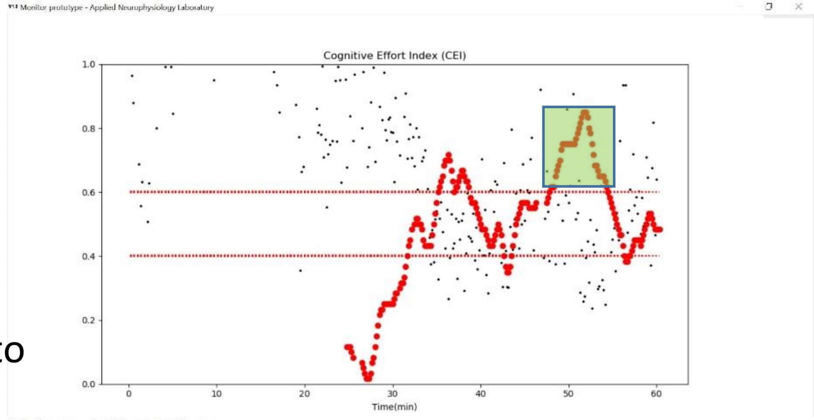

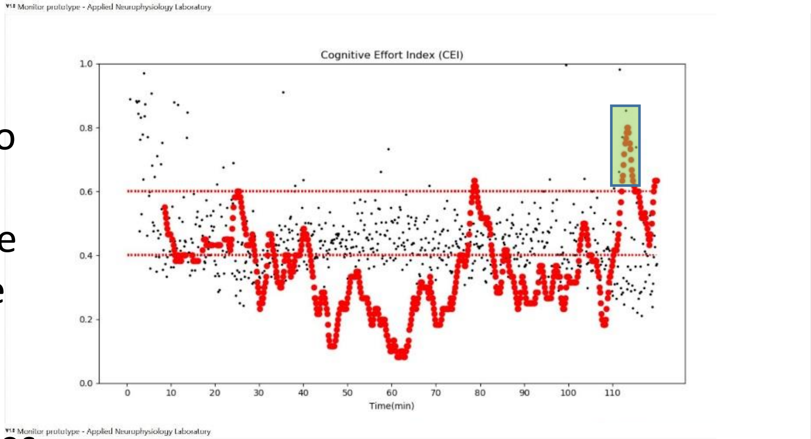

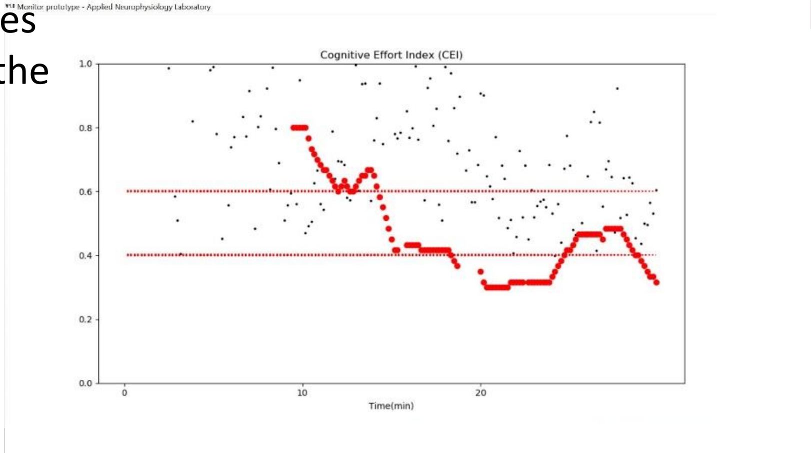

Recall

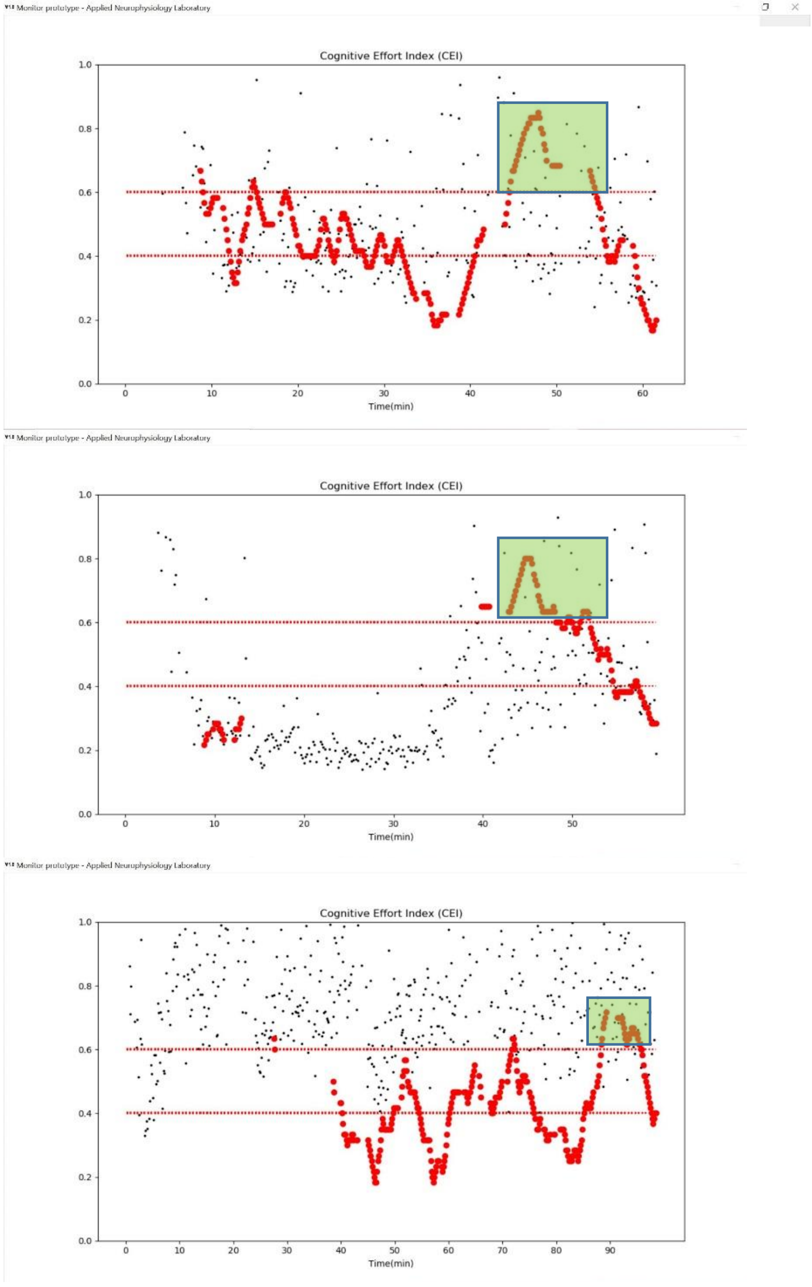

No recall

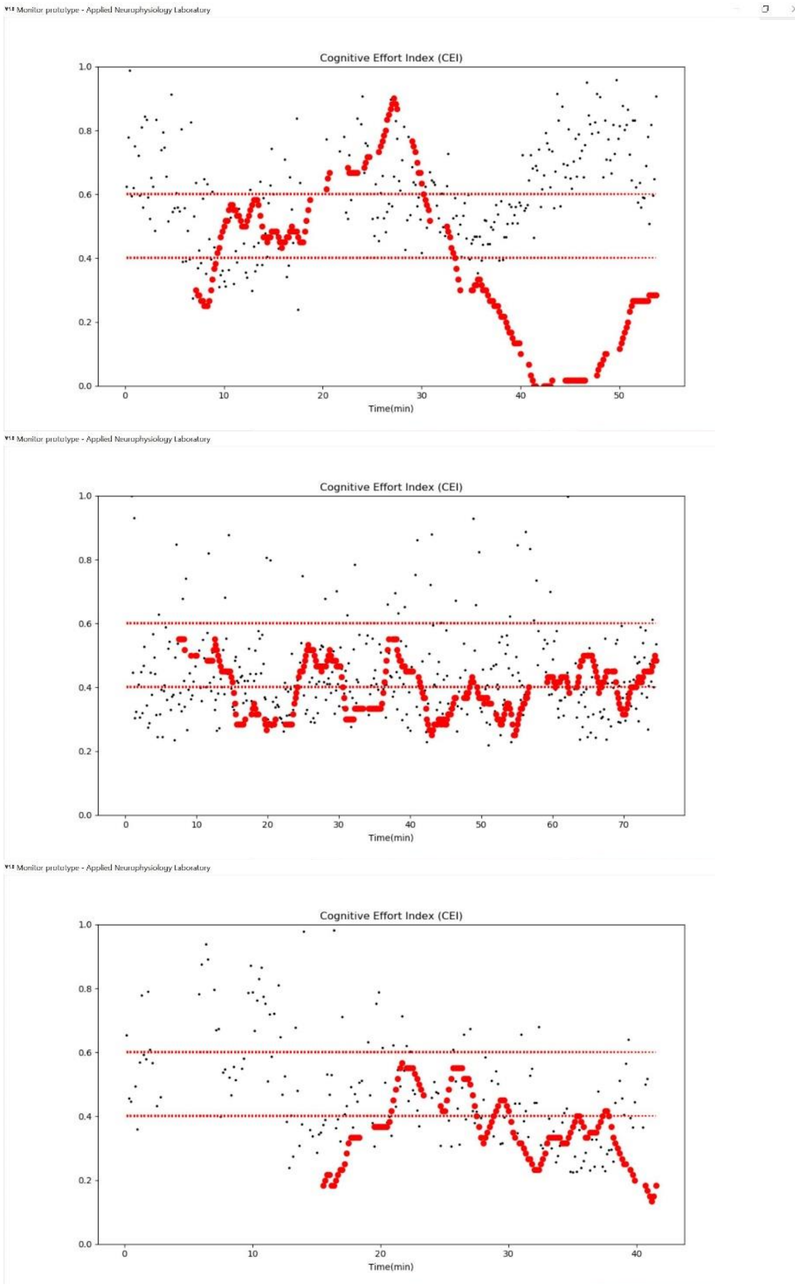

Recall

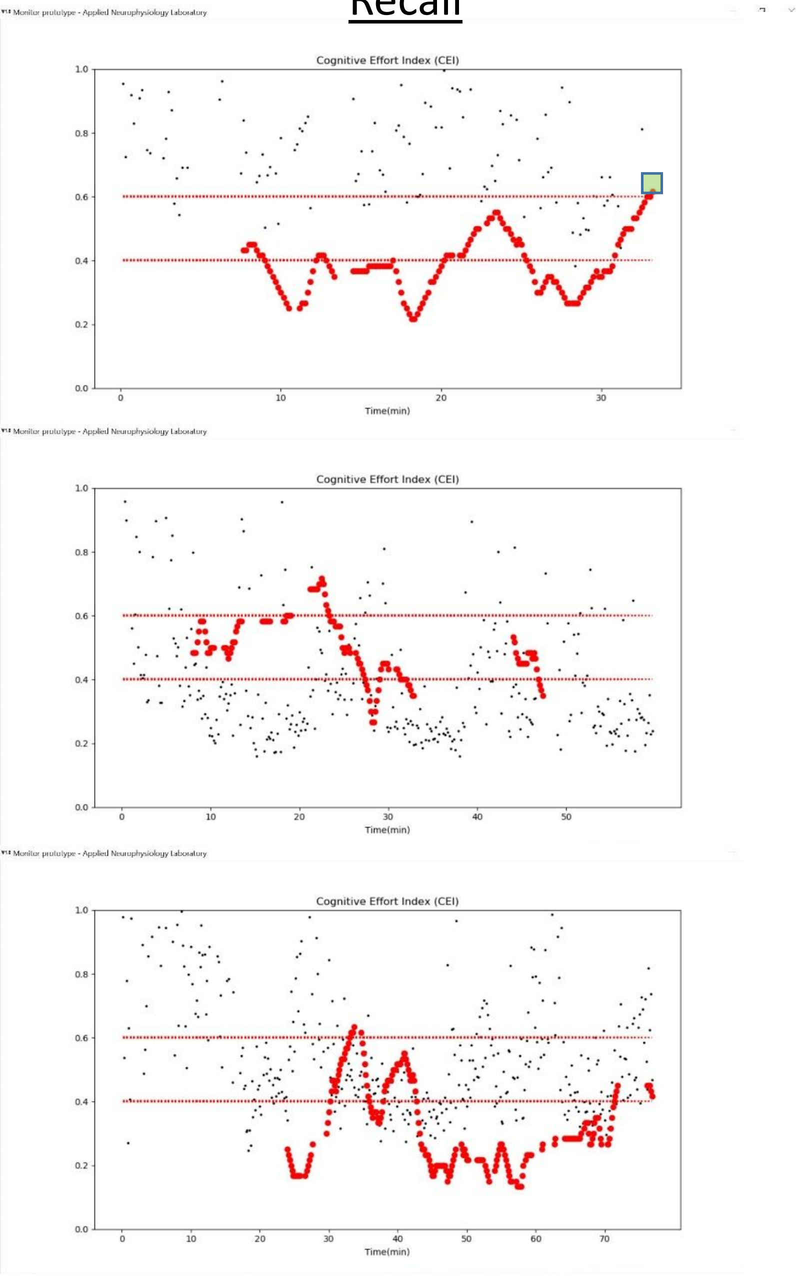

No recall

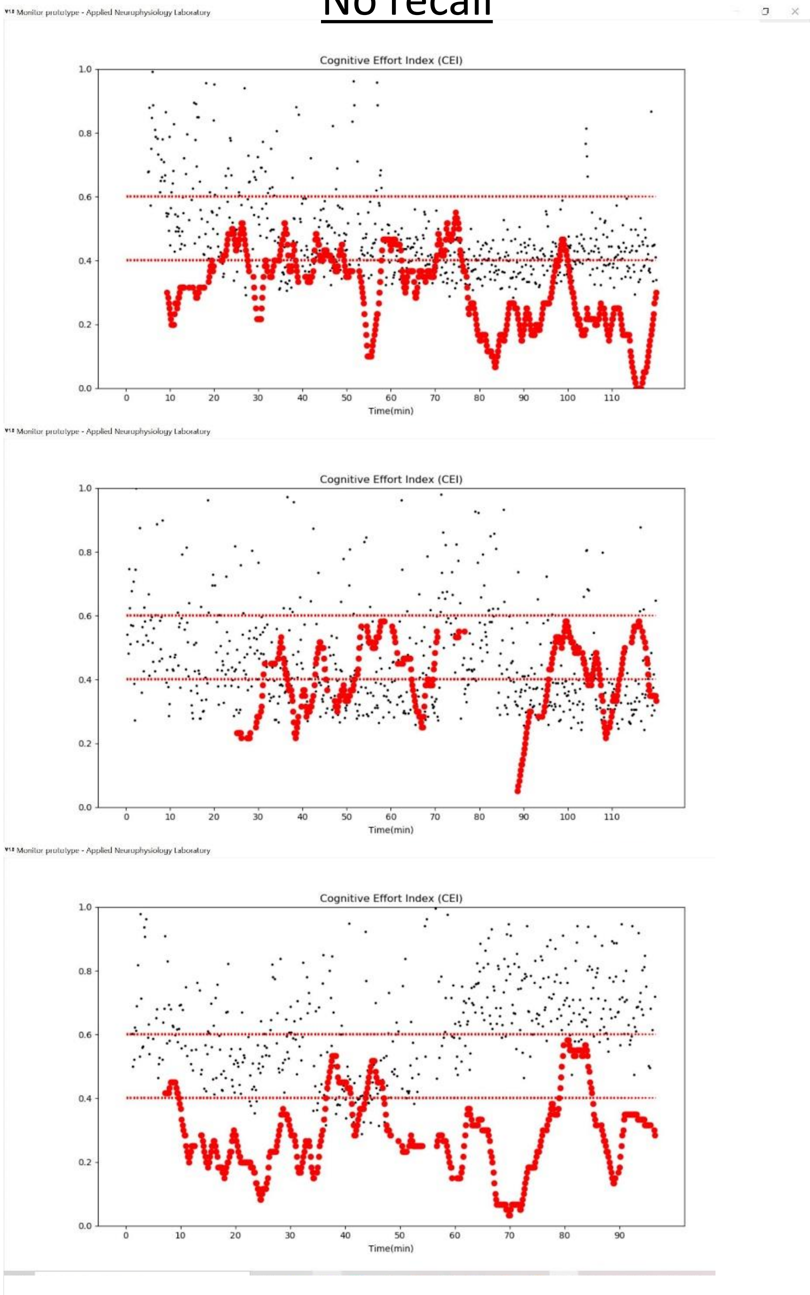

Recall

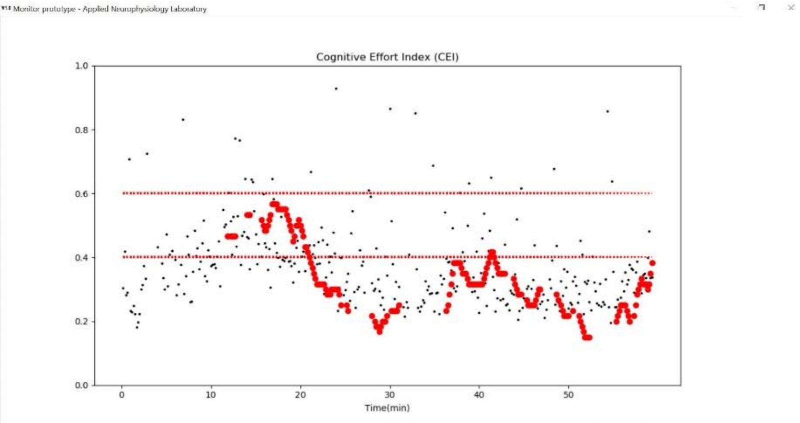

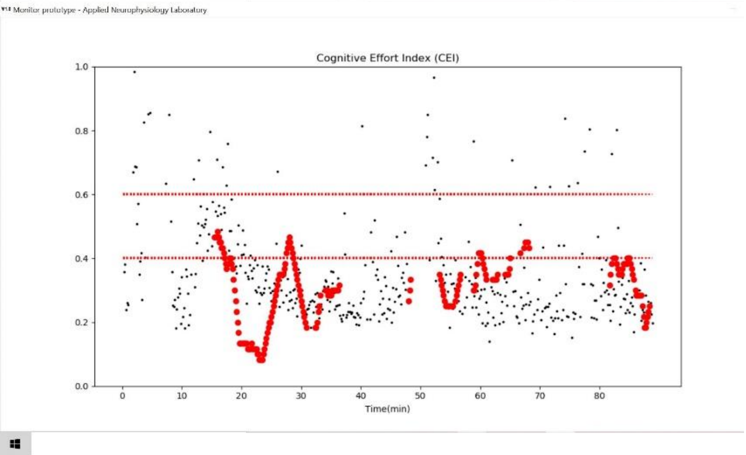

No recall

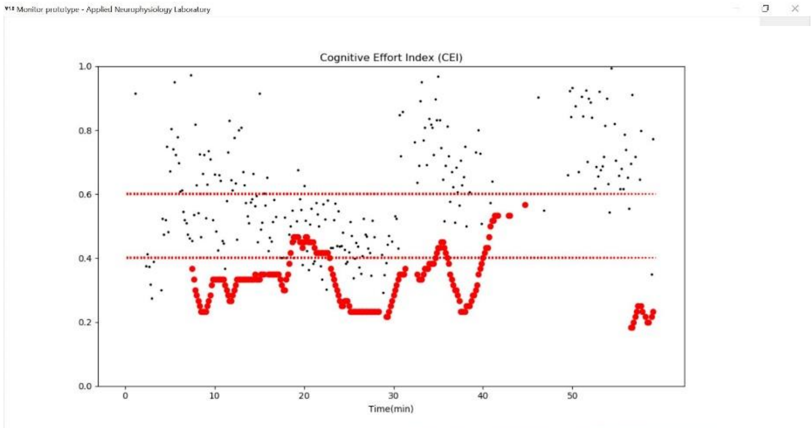

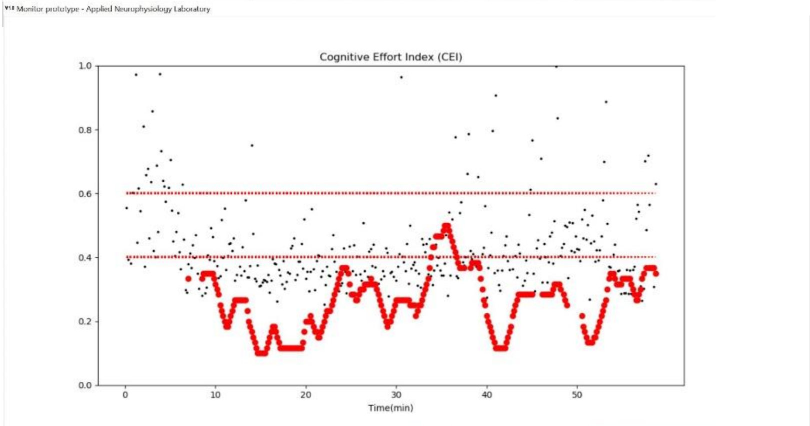

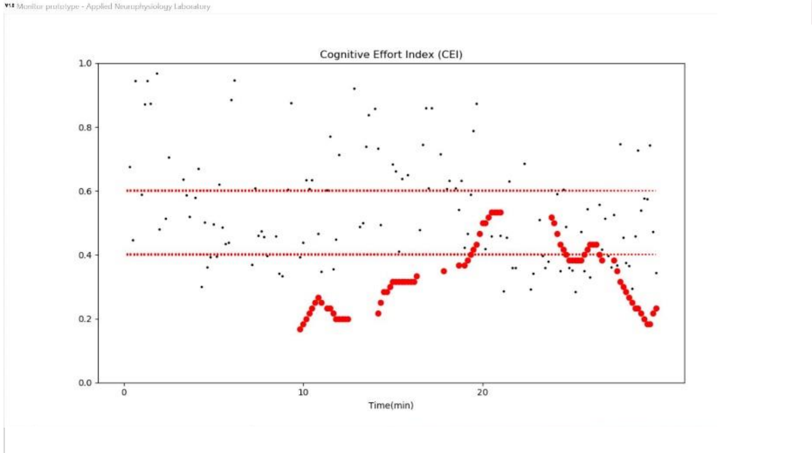

No recall

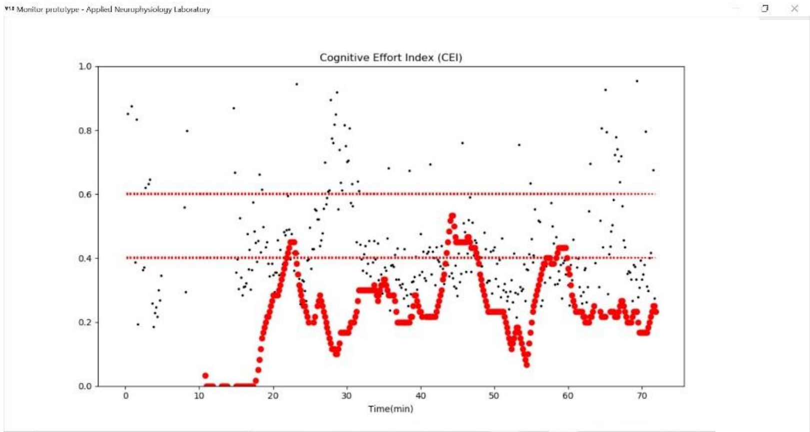
Monitor prototype - Applied Neurophysiology Laboratory
Cognitive Effort Index (CEI)
1.0
0.8
0.6
0.4
0.2
0.0
0
10
20
30
40
50
60
70
Time(min)

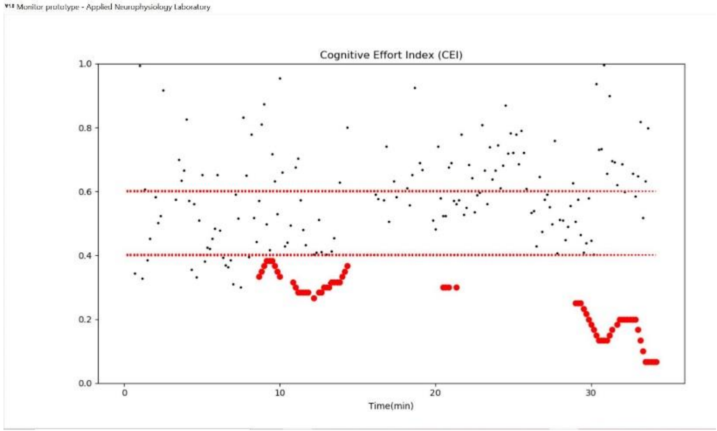
Monitor prototype - Applied Neurophysiology Laboratory
Cognitive Effort Index (CEI)
1.0
0.8
0.6
0.4
0.2
0.0
0
10
20
30
Time(min)
