# Supplementary material for: Identifying Recall Under Sedation by a Novel EEG Based Index of Attention—A Pilot Study
Source: Front Med (Lausanne). 2022 Apr 14;9:880384. doi: 10.3389/fmed.2022.880384 (PMC9047181; doi:10.3389/fmed.2022.880384)
Supplement: Supplementary file 2 [file Data_Sheet_2.PDF]

A. Cardiac surgery

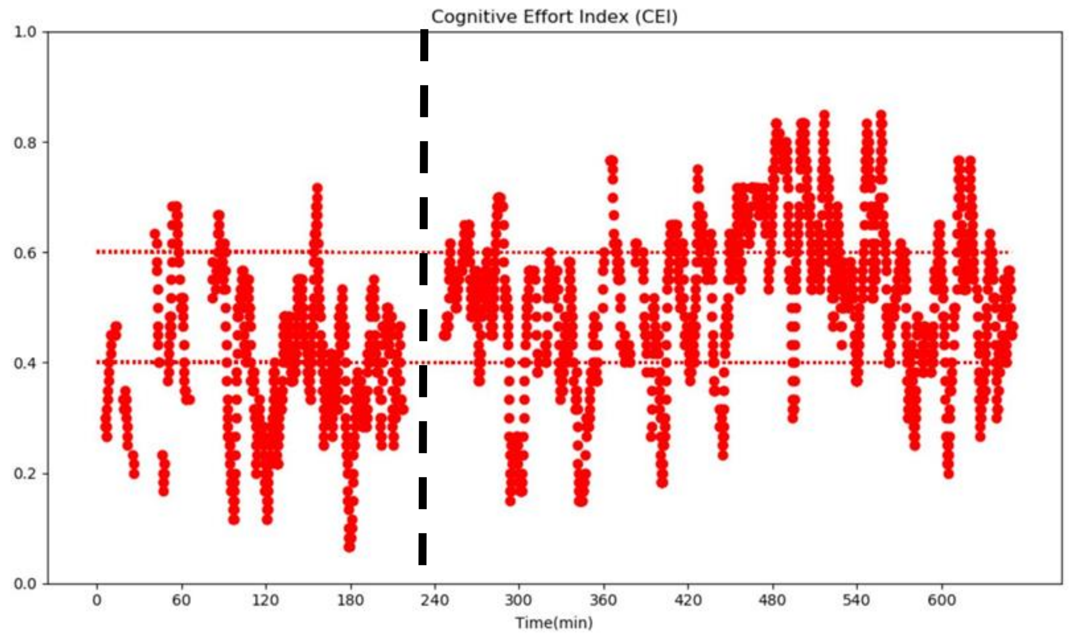

B. Orthopedic surgery

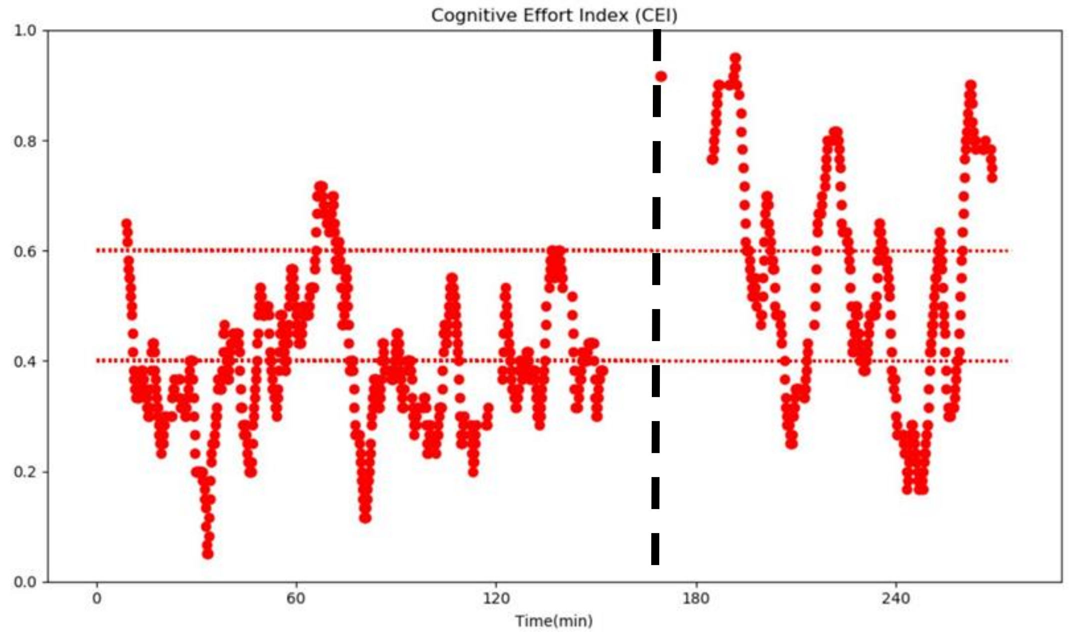

CEIrc dynamics in two patients during the intraoperative and early postoperative periods (without assessment of awareness under anesthesia). The end of surgery is marked by the dashed black line. Patient A underwent elective cardiac surgery and was completely awake some four hours after surgery. Patient B underwent elective orthopedic surgery under sedation and spinal anesthesia and was awakened immediately after surgery.

The sample was done with BIS Medtronic, with the EEG sample downloaded to disk-on-key for offline analysis. Missing points represent automatic rejection of noisy sample periods.
